# Less Fluctuation in Hemodynamics of the Wide-Awake Local Anesthesia No Tourniquet Technique Than General Anesthesia in Distal Radius Plating Surgery: A Prospective Case-Control Study

**DOI:** 10.3390/jcm11041123

**Published:** 2022-02-21

**Authors:** Wen-Chih Liu, I-Cheng Lu, Chung-Chia Chang, Chih-Ting Chen, Chung-Hwan Chen, Chia-Lung Shih, Yin-Chih Fu, Jesse Bernard Jupiter

**Affiliations:** 1 Department Orthopedic Surgery, Kaohsiung Medical University Hospital, Kaohsiung Medical University, Kaohsiung 80756, Taiwan; andysirliu@gmail.com (W.-C.L.); luckychungchia@gmail.com (C.-C.C.); hwan@kmu.edu.tw (C.-H.C.); 2 Department of Orthopedic Surgery, Kaohsiung Municipal Siaogang Hospital, Kaohsiung Medical University, Kaohsiung 81267, Taiwan; 3 Ph.D. Program in Biomedical Engineering, College of Medicine, Kaohsiung Medical University, Kaohsiung 80756, Taiwan; 4 Regeneration Medicine and Cell Therapy Research Center, Kaohsiung Medical University, Kaohsiung 80756, Taiwan; 5 Department of Anesthesiology, Kaohsiung Municipal Siaogang Hospital, Kaohsiung Medical University, Kaohsiung 81267, Taiwan; u9251112@gmail.com; 6 School of Medicine, College of Medicine, Kaohsiung Medical University, Kaohsiung 80756, Taiwan; 7 School of Post-Baccalaureate Medicine, College of Medicine, Kaohsiung Medical University, Kaohsiung 80756, Taiwan; u107000041@gap.kmu.edu.tw; 8 Department of Orthopedic Surgery, Kaohsiung Municipal Ta-Tung Hospital, Kaohsiung 80145, Taiwan; 9 Department of Medical Research, Ditmanson Medical Foundation Chiayi Christian Hospital, Chiayi 60002, Taiwan; stone770116@gmail.com; 10 Hand and Arm Center, Department of Orthopedic Surgery, Massachusetts General Hospital, Harvard Medical School, Boston, MA 02114, USA; jjupiter1@partners.org

**Keywords:** WALANT, wide-awake local anesthesia no tourniquet, wide-awake hand surgery, distal radius fracture, balanced anesthesia

## Abstract

This prospective case-control study aimed to compare the intraoperative hemodynamic changes between the wide-awake local anesthesia no tourniquet (WALANT) technique and general anesthesia (GA) in patients undergoing distal radius plating surgery. Forty adults with distal radius fractures underwent plating surgery via the WALANT technique (20 patients) or GA (20 patients). Mean arterial pressure (MAP) and heart rate were recorded. Intraoperative pain intensity was measured using the visual analog scale (VAS) for pain in the WALANT group. The measures of hemodynamics and VAS were recorded at seven-time points perioperatively. The VAS score decreased significantly compared with the preoperative status in the WALANT group for most of the intraoperative period except during injections of local anesthetics and fracture reduction. The intraoperative MAP in the WALANT group showed no significant change during the perioperative period. In addition, the WALANT group showed fewer perioperative MAP fluctuations than the GA group (*p* < 0.05). The reduction and plating quality were similar between the two groups. WALANT provided a feasible technique with less fluctuation in hemodynamic status. With gentle manipulation of the fracture reduction, distal radius plating surgery using the WALANT technique is a well-tolerated surgical procedure and shows similar reduction and plating quality to GA.

## 1. Introduction

Wide-awake local anesthesia no tourniquet (WALANT) is a promising technique for performing various hand surgeries [1,2]. This technique employs a large amount of local anesthetic with epinephrine over the surgical site. Under the hemostatic effect of epinephrine, a relatively bloodless surgical field can be created without the use of a tourniquet. Because of the nature of tumescent local anesthesia, local anesthetic injections around the nerve are unnecessary and increase the risk of nerve injury [3]. WALANT offers technical simplicity, and a surgeon can perform the entire procedure without the use of sedation.

Due to the advantages of WALANT over GA, the technique has become popular for various hand/upper extremity procedures in recent decades. Hand and upper extremity surgery using the WALANT technique may mitigate the use of opioids [4] and earlier analgesic stoppage [5]. The WALANT technique is optimal for the urgent treatment of hand infections [6]. In addition, the infection risk did not increase in the carpal tunnel or trigger finger release using the WALANT technique [7]. In recent years, the WALANT technique has been employed for bony procedures, such as open reduction plating for distal radius fractures [5,8,9,10,11], olecranon fractures [12], and ankle fractures [13]. Because there are no major complications, previous studies have concluded that the WALANT technique is safe and cost-effective for distal radius plating surgery [10,12,14]. Comparing the surgical outcomes in distal radius fracture plating surgery using the WALANT technique and GA, similar functional outcomes but less postoperative pain were found [9,15,16]. Thus, the WALANT technique appears to be more attractive than GA.

Concerning intraoperative pain during WALANT distal radius plating surgery, some surgeons still hesitate to adopt this technique because of concerns regarding patients’ agitation and physiological responses to pain in bony procedures, such as distal radius plating surgery. A study showed that WALANT distal radius plating surgery has similar functional outcomes and no increase in pain level compared with brachial plexus block (BPB) [17]. A clinical trial comparing distal radius plating surgery using WALANT and GA showed that those treated by the WALANT technique had no difference in perioperative anxiety level and intraoperative visual analog scale for pain (VAS) [15]. Although hand surgery via the WALANT technique showed tolerable pain and optimal outcomes, patients still have concerns about feeling pain [18]. As a result, more evidence is needed to confirm the detailed intraoperative physiological changes and pain scale of patients who underwent surgery using the WALANT technique.

This prospective case-control study aimed to compare the hemodynamic changes in patients undergoing distal radius plating using two different anesthesia methods: WALANT and general anesthesia (GA). Our primary outcome was the patient’s mean arterial pressure (MAP) and heart rate (HR). The secondary outcome was intraoperative VAS when using the WALANT technique. We also compared the quality of the perioperative plating in each group. The objective physical response to pain appeared with changes in MAP and HR [19]. We hypothesized that changes in MAP and HR might be smaller in the WALANT group. This study aimed to evaluate whether the WALANT technique provides a relatively stable hemodynamic response during distal radius fracture plating surgery.

## 2. Materials and Methods

From January 2019 to February 2020, 40 adults with DRF undergoing open reduction and plating surgery at a standard operation theater of a university-affiliated hospital were recruited. Patients with multiple injuries, an open fracture, or a pathological fracture were excluded. This prospective observational study was approved by the Institutional Review Board of Kaohsiung Medical University Hospital (KMUHIRB-F(I)-20180116). The mean age of the patient was 61 ± 14 years old. Eleven patients had diabetes mellitus, 13 patients had hypertension, three patients had a history of a cerebral vascular accident, two patients had chronic kidney disease and one patient had chronic heart failure, and two patients had evidence of vascular calcification in the wrist and hand vessels. Regarding the anesthetic risk of the included patients, 25 patients were classified as American Society of Anesthesiologists physical status (ASA) 2, 14 patients were classified as ASA 3, and one patient was classified as ASA 4. Patients with multiple injuries, an open fracture, or a pathological fracture were excluded. The indication for undergoing WALANT or general GA depended on the patients’ preference. Patients in the WALANT group underwent surgery using the WALANT technique, and all surgical procedures and administration of local anesthetics were performed by the same surgeon.

In the WALANT group, following the conservative upper limit of 7 mg/kg in lidocaine injection [3], 1% lidocaine with 1:100,000 epinephrine was used. For a person weighing 60 kg, this equates to 420 mg or 42 mL of 1% lidocaine could be used. Patients’ forearms were placed in a supine position. Local anesthetic injection began with employing the Henry approach to make an incision at the proximal end with a 26-G needle. After 2–3 mL had been injected into the subcutaneous fat, the 26-G needle was exchanged with a 22-G long needle, and the local anesthetic was slowly injected along the volar incision wound from the same entry point. Typically, 15 mL is enough to cover all the volar surface of the subcutaneous area for Henry approach incisions. A 24 G needle was then used through the pronator quadratus and touched the volar surface of the radius; 10 mL of local anesthetic was injected into the fracture site and along the distal radius volar periosteum. Patients pronated their forearm, and 10 mL of local anesthetic was injected along the dorsal periosteum. Finally, 2–3 mL was injected over the radial styloid to prepare for preliminary K-wire fixation. The local anesthetic injection procedure generally took 5–10 min to perform. We prepped after the injection of local anesthetic. Surgery was performed 20 min after local anesthetic injection, once the hemostatic effect of epinephrine was observed.

In the GA group, patients underwent surgery using GA with ultrasound-guided brachial plexus block (BPB) at the supraclavicular level. Balanced anesthesia and multimodal analgesia are the current standard approach in our institute. GA was induced with 1 mcg/kg fentanyl and 2 mg/kg propofol and maintained with 2–4% sevoflurane. Premedication, including benzodiazepine or opioids, was not in our routine practice. The anesthesiologist identified the ultrasound image of the brachial plexus at the supraclavicular level. Under real-time ultrasound guidance, the anesthesiologist performed a supraclavicular block with 25 mL of 0.25% bupivacaine. Thereafter, a tourniquet was applied to the patient’s upper arm. After exsanguinating the forearm blood with an Esmach bandage, the tourniquet was set up at 250 mmHg and the surgery for DRF was performed.

GA was induced by two experienced anesthesiologists, and the surgical procedure was performed by three surgeons. All surgeons performing the surgical procedures were classified as having level 3 expertise [20]. Different volar locking plates (ACU-LOC plate, ACUMED, LLC., Hillsboro, OR, USA; Anatomic Volar Plate System, Depuy Synthes, Johnson & Johnson Co., New Brunswick, NJ, USA; Distal R.A.F. Locking plate, APLUS Co., Taiwan, China) were used for internal fixation in all cases. A standard volar approach was used to expose the fractured side. The fracture was approached from the radial side of the flexor carpi radialis, and the quadrate pronator muscle was incised to reduce the fracture.

In the WALANT group, MAP, HR, and VAS were measured by nursing staff in the operation theatre seven times perioperatively, namely before surgery (T0) and at the time of injection of local anesthesia (T1), skin incision (T2), fracture reduction (T3), plating and screwing (T4), skin closure (T5), surgery completion (T6). In the GA group, the anesthesia team continuously monitored patients’ intraoperative physiological status. MAP and HR in the GA group were marked after induction (T1) and at the other six same time points as in the WALANT group.

We recorded the operative time of each group, from skin incision to wound closure. The blood loss was recorded in each group. In the GA group, the blood loss was recorded after wound closure and before tourniquet deflation. The radiographic parameters for examining the preoperative and postoperative quality of reduction and plating including radial height, radial inclination, volar tilt, ulnar variance, and articular step-offs were compared between each group by a qualified orthopedic resident and confirmed by another senior hand surgeon. The radiographic parameters criteria were based on classic literature [21]. The radial inclination and ulnar variance were measured on the wrist posteroanterior (PA) view. The volar tilt was measured on the lateral view. The articular step-off was determined on the largest articular gap on either PA or lateral view. Anesthesia-related (and surgery-related) risk within 30 days of surgery was also recorded.

The data in this study were examined by an independent operator using descriptive statistics. Continuous variables were expressed as the mean and standard deviation, and categorical variables were expressed as the total number of events. Fisher’s exact test was used to analyze the categorical data. After assessing the normal distribution, repeated measures ANOVA were used for comparing MAP and HR within and between treatment groups, and the means of absolute changes in MAP and HR among treatment groups. Paired *t*-tests were used to compare radial inclination, radial height, and ulnar variance. Depending on the nonnormal distribution, the Wilcoxon singed-rank test was used for comparing VAS from T1 to T6 and T0. The Mann-Whitney U test was used to compare the articular step-offs. A two-tailed *p* < 0.05 was considered statistically significant. Statistical analysis was performed using SPSS v26 (IBM, Armonk, NY, USA).

Due to the lack of preliminary results that compare intraoperative hemodynamics between the WALANT technique and GA in distal radius fracture plating surgery, 20 patients in each group were included based on a previous study that compared two types of anesthesia in the same surgery [22]. A post-hoc power analysis was conducted using G*Power (version 3.1.9.6) [23,24]. A significance two-tail threshold was set at *p* = 0.05, and the mean difference and standard deviation (SD) of the intraoperative MAP and preoperative MAP in both groups were calculated for the effect size (Cohen’s d), and the number in each group was 20. Under these circumstances, the post-hoc power was calculated.

## 3. Results

We recruited 40 patients who underwent distal radius plating surgery, and 20 patients were equally distributed to the WALANT and GA groups. The demographic information, comorbidity, DRF pattern, surgical time, and amount of blood loss in each group are presented in Table 1. The intraoperative VAS in The WALANT group demonstrated a statistically significant decrease from T0 to T2, T4, T5, and T6 (*p* < 0.05). However, there was no significant difference between T0 versus T1 (2.30 ± 2.43 versus 1.90 ± 2.38, *p* = 0.514) and T0 versus T3 (2.30 ± 2.43 versus 1.80 ± 2.35, *p* = 0.567) (Table 2).

**Figure 1:**
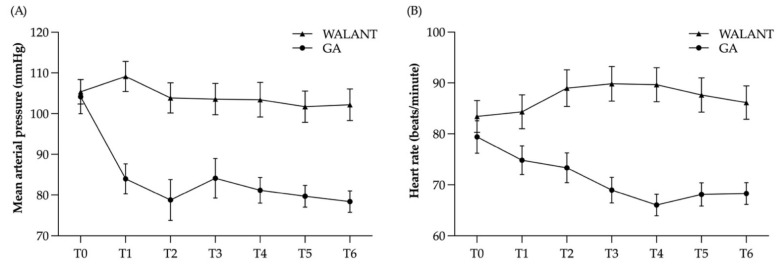
Graphs showing the perioperative changes in mean arterial pressure (MAP) and heart rate (HR). (**A**) Preoperative MAP (T0) exhibited no statistically significant differences between the wide-awake local anesthesia no tourniquet (WALANT) group and the general anesthesia (GA) group. MAP in the GA group dropped after the induction of anesthesia. The WALANT group showed less fluctuation in MAP than the GA group. (**B**) The preoperative HR showed no statistically significant differences between the two groups. HR exhibited a mild increase in the WALANT group and a greater decrease in the GA group. The WALANT group showed less fluctuation in HR than the GA group. The error bars represent standard error of the mean.

The perioperative MAP and HR are displayed in Figure 1. The preoperative MAP and HR exhibited no statistically significant differences between the groups (Table S1). The change of MAP between T1 to T0 was significantly lower in The WALANT group than in the GA group (3.77 ± 9.32 versus −20.46 ± 14.16, *p* < 0.001). The WALANT group showed less variability in MAP and HR than the GA group perioperatively (Table 3). The post-hoc calculation of the effect showed a Cohen’s d of 2.02, and the post-hoc power was 99.9%.

The MAP showed no difference (*p* = 0.068) among the WALANT group but a statistically significant difference among the GA group (*p* < 0.001). Further analyses with post-hoc pairwise comparisons, the MAP among the WALANT group showed no significant difference at T0 than that at T1 to T6. However, the MAP among the GA group was shown to be significantly greater at T0 than that at T1 to T6 (Tables S2 and S3).

The reduction and plating quality were similar between each group in preoperative and postoperative radiograph (Table 4). There were no perioperative cardiovascular events or postoperative events of infection, neuropraxia, or compartment syndrome. A transient bluish appearance distal to the injected area was frequently noted in the WALANT group. It may be the result of a mechanical block of venous return by a tumescent local anesthetic. However, a 39-year-old woman had histories of psoriasis vulgaris and cold sensitivity in the hands. No vascular calcification was noted in her hand and wrist vessels. After the injections of local anesthetic, a transient vascular compromise event occurred. However, no permanent complications were noted during follow-up [25].

## 4. Discussion

WALANT is a promising approach that hand surgeons can adopt to perform different types of soft tissue and bony procedures. Recent studies [9,10] have reported its safety and favorable postoperative outcomes when used for open reduction and plating for DRF. In a cohort study [16] comparing distal radius plating surgery using the WALANT technique to GA, the WALANT group showed similar blood pressure and tolerable intraoperative pain (14/20 reported no pain, 6/20 reported VAS ≤ 3). In our study, a detailed pain scale at seven-time points during the operation was provided. The difference between our study and previous studies is that we recorded seven-time points of interoperative VAS, MAP, and HR to determine the detailed hemodynamics in each period during the surgery. The results of our study indicate that a relatively stable intraoperative physiological status was noted in patients undergoing surgery using WALANT than in those undergoing GA. Compared to the preoperative pain status, the patient showed mild discomfort during injection of local anesthetics and fracture reduction and was almost pain-free in the other intraoperative periods.

Pain is an unpleasant sensory and emotional experience associated with actual and potential tissue damage. VAS is the most frequently used method to describe pain severity. VAS ranges from “0,” representing no pain, to “10,” representing the worst pain imaginable. Objective pathophysiological response to nociceptive stimuli is also called nociception. Many commercial monitoring methods have been proposed, and the analgesia nociception index (ANI) is one of the parameters based on the high-frequency component of heart rate variability [26]. In addition, changes in ANI paralleled the changes in HR and MAP [19]. Because the ANI monitor is not available everywhere, we could indirectly testify to the patient’s nociception level by the changes in MAP and HR.

When we injected a local anesthetic into the broken forearm, administration of 40 mL of a local anesthetic around the wrist led to patient discomfort due to wrist swelling. All patients were informed that the bulging was normal and should not be painful. Some patients tolerated this effect, but others could not. However, the patients always felt bulging relief after the skin incision. The VAS at T1 was not significantly different from that at T0 in the WALANT group, and the MAP between T0 and T1 showed no significant difference. The significant drop in mean MAP from T0 to T1 using GA was obviously caused by the sedative effect of the anesthetics. Since the blood loss in both groups was small, we could attribute the better intraoperative hemodynamic control to the fewer systemic effects of the local anesthetic and less physical response to intraoperative noxious stimuli, for example, drilling holes for driving screws or manipulation of the fragments for fracture reduction.

The most uncomfortable timepoint for patients is during the period of fracture reduction. The technique that surgeons adopted to perform this step smoothly was to provide adequate hematoma block (i.e., an appropriate amount of local anesthetic) to the fracture site to infiltrate the periosteum surrounding the dorsal and volar cortex. We inserted the needle radial to the flexor carpi radialis to prevent injury to the median nerve. Patients occasionally felt uncomfortable during the reduction, as indicated by the insignificant reduction in VAS scores. However, we did not observe that the changes in MAP were significantly larger than those observed when applying GA.

Although the amount of intraoperative blood loss was higher in the WALANT group than in the GA group (14.15 mL vs. 6.55 mL, *p* = 0.008), we considered it not clinically different between both groups. In this study, the amount of blood loss was calculated before deflation of the pneumatic tourniquet. Under the local hemostatic effect of epinephrine and careful electrocoagulation, the amount of blood loss was very limited and did not interfere with the surgical procedure. Without the use of a tourniquet, we could feel and see the pulsation of the radial artery, which enabled us to coagulate some small vessels more confidently by using WALANT. Moreover, the higher MAP in the WALANT group might have contributed to higher blood loss.

Although the basic patient characteristics between the two groups were similar, surgeon preference and selection bias may still exist in this nonrandomized study. Although the fracture characteristics in each group showed no difference between the groups, we preferred not to use the WALANT technique with highly comminuted and unstable fractures, which might require complex reduction techniques and multiple modalities for fixation. We suggest that WALANT is not indicated for patients who are intolerant to lying straight. As a result, we have no experience in ceasing surgeries undertaken with the WALANT technique due to intraoperative intolerance, even though some patients were anxious and expressed their fear of pain intolerance preoperatively. An adequate local anesthetic may provide sufficient analgesic effect in performing the surgery.

There are several limitations to our study. First, although there were 20 patients in each group, which is sufficient according to a previous study [22], and the pos-hoc power was checked, the sample size was still small. In addition, WALANT is a relatively new technique for distal radius fracture plating, and we believe it is worthwhile to provide our results and encourage others to conduct the same clinical research and confirm our results [27]. Second, this was a non-randomized, prospective, observational study. Some selection bias might exist; for example, patients with higher anxiety and lower intolerance to pain might choose GA. However, most of the patients’ preoperative demographics, including fracture patterns, demonstrated no differences between the two groups. Third, data collection bias exists as the study staff in the operating theater knew which group the patients were allocated to. Finally, every anesthesiologist has personal preferences regarding the mode of administration (inhaled and intravenous) of anesthetics for GA, and the quality of ultrasound-guided BPB might vary among anesthesiologists.

## 5. Conclusions

Distal radius plating surgery using the WALANT technique is a well-tolerated surgical procedure and shows similar reduction and plating quality to that of GA. WALANT provided a feasible technique with less fluctuation in hemodynamic status. WALANT could be an alternative when GA is high risk or unavailable.

## Figures and Tables

**Table 1:** Demographic data.

	WALANT	GA	*p*-Value
Patient number	20	20	
Age (year)	62.2 ± 13.5	59.9 ± 14.6	0.718
Sex			
Male	3	6	0.256
Female	17	14	
ASA			
2	12	13	0.595
3	7	7	
4	1	0	
Comorbidity			
Diabetes Mellitus	6	5	0.723
Hypertension	5	8	0.311
Cerebral vascular accident	1	2	0.548
Chronic kidney disease	0	2	0.147
Chronic heart failure	1	0	0.311
Forearm and wrist vascular calcification	1	1	1.000
Fracture classification			
AO 2R3A	14	11	0.606
AO 2R3B	3	5	
AO 2R3C	3	4	
Operation time (minute)	57.8 ± 16.4	63.8 ± 20.6	0.512
Blood loss (mL)	14.2 ± 13.1	6.6 ± 5.9	**0.008**
Complication			
Infection	0	0	-
Neuropraxia	0	0	-
Compartment syndrome	0	0	-
Perioperative cardiovascular event	0	0	-
Hand vascular compromise event	1	0	0.331
Exchange to general anesthesia	0	-	-

WALANT, wide-awake local anesthesia no tourniquet; GA, general anesthesia; ASA, American Society of Anesthesiologists’ classification; mean ± standard deviation. Bold means *p*-value < 0.05.

**Table 2:** Perioperative visual analog scale for pain.

	Mean	Standard Deviation	*p*-Value
T0	2.30	2.43	
T1	1.90	2.38	0.514
T2	0.60	0.88	**0.011**
T3	1.80	2.35	0.567
T4	0.85	1.18	**0.033**
T5	0.40	0.75	**0.003**
T6	0.40	0.75	**0.003**

Bold means *p*-value < 0.05.

**Table 3:** Perioperative mean arterial pressure and heart rate variabilities.

	WALANT		GA		*p*-Value
	Mean	SD	Mean	SD	
∆MAP					
T1–T0	3.77	9.32	−20.46	14.16	**<0.001**
T2–T1	−5.28	6.83	−5.76	19.23	0.916
T3–T2	−0.27	4.39	4.92	13.74	0.113
T4–T3	−0.17	5.52	−2.27	12.06	0.481
T5–T4	−1.72	6.42	−1.71	8.32	0.999
T6–T5	0.47	6.66	−1.29	5.25	0.354
∆HR					
T1–T0	0.90	8.41	−4.29	10.24	0.085
T2–T1	4.65	8.82	−2.43	11.90	**0.037**
T3–T2	0.85	4.49	−3.67	6.95	**0.018**
T4–T3	−0.20	2.04	−2.14	5.44	0.139
T5–T4	−2.00	6.67	1.67	6.44	0.081
T6–T5	−1.50	3.95	−0.10	4.54	0.298

WALANT, wide-awake local anesthesia no tourniquet; GA, general anesthesia; SD, standard deviation; ∆MAP, change in mean arterial pressure; ∆HR, change in heart rate. Bold means *p*-value < 0.05.

**Table 4:** Radiologic measurements comparing the WALANT group to GA group preoperative and postoperative values.

	Pre-Surgery			Post-Surgery		
	WALANT	GA	*p*-Value	WALANT	GA	*p*-Value
RI (°)	20.8 ± 5.1	17.0 ± 7.2	0.840 ^a^	23.1 ± 3.4	19.9 ± 4.5	0.023 ^a^
UV (mm)	3.5 ± 2.1	2.5 ± 3.5	0.314 ^a^	0.6 ± 1.7	−0.4 ± 2.3	0.153 ^a^
RH (mm)	8.5 ± 2.5	8.4 ± 3.7	0.940 ^a^	10.5 ± 1.6	10.1 ± 2.6	0.649 ^a^
ASO (mm)	0.4 ± 0.7	0.3 ± 0.5	0.983 ^b^	0.0 ± 0.0	0.1 ± 0.2	0.344 ^b^
VT (°)	−12.0 ± 18.4	−6.0 ± 15.4	0.296 ^a^	7.4 ± 6.0	4.8 ± 6.3	0.221 ^a^

RI, radial inclination; UV, ulnar variance; RH, radial height; ASO, articular step-off; VT, volar tilt; ^a^: paired *t*-test; ^b^: the Mann–Whitney U test; mean ± standard deviation.

## Data Availability

The data presented in this study are available in article and supplementary material.

## Supplementary Materials

The following supporting information can be downloaded at: https://www.mdpi.com/2077-0383/11/4/1123/s1, Table S1: Perioperative mean arterial pressure and heart rate; Table S2: Pairwise comparisons in MAP and HR among WALANT group; Table S3: Pairwise comparisons in MAP and HR among GA group.
