# Correction: Liu et al. Less Fluctuation in Hemodynamics of the Wide-Awake Local Anesthesia No Tourniquet Technique Than General Anesthesia in Distal Radius Plating Surgery: A Prospective Case-Control Study. *J. Clin. Med.* 2022, *11*, 1123

**DOI:** 10.3390/jcm11123381

**Published:** 2022-06-13

**Authors:** Wen-Chih Liu, I-Cheng Lu, Chung-Chia Chang, Chih-Ting Chen, Chung-Hwan Chen, Chia-Lung Shih, Yin-Chih Fu, Jesse Bernard Jupiter

**Affiliations:** 1 Department Orthopedic Surgery, Kaohsiung Medical University Hospital, Kaohsiung Medical University, Kaohsiung 80756, Taiwan; andysirliu@gmail.com (W.-C.L.); luckychungchia@gmail.com (C.-C.C.); hwan@kmu.edu.tw (C.-H.C.); 2 Department of Orthopedic Surgery, Kaohsiung Municipal Siaogang Hospital, Kaohsiung Medical University, Kaohsiung 81267, Taiwan; 3 Ph.D. Program in Biomedical Engineering, College of Medicine, Kaohsiung Medical University, Kaohsiung 80756, Taiwan; 4 Regeneration Medicine and Cell Therapy Research Center, Kaohsiung Medical University, Kaohsiung 80756, Taiwan; 5 Department of Anesthesiology, Kaohsiung Municipal Siaogang Hospital, Kaohsiung Medical University, Kaohsiung 81267, Taiwan; u9251112@gmail.com; 6 School of Medicine, College of Medicine, Kaohsiung Medical University, Kaohsiung 80756, Taiwan; 7 School of Post-Baccalaureate Medicine, College of Medicine, Kaohsiung Medical University, Kaohsiung 80756, Taiwan; u107000041@gap.kmu.edu.tw; 8 Department of Orthopedic Surgery, Kaohsiung Municipal Ta-Tung Hospital, Kaohsiung 80145, Taiwan; 9 Department of Medical Research, Ditmanson Medical Foundation Chiayi Christian Hospital, Chiayi 60002, Taiwan; stone770116@gmail.com; 10 Hand and Arm Center, Department of Orthopedic Surgery, Massachusetts General Hospital, Harvard Medical School, Boston, MA 02114, USA; jjupiter1@partners.org

In the original publication [1], there was a mistake in Table 1. The subclassifications of the fracture classifcation were disordered, as follows:

The following table has been modified:

**Table 1:** Demographic data.

	WALANT	GA	*p*-Value
Patient number	20	20	
Age (year)	62.2 ± 13.5	59.9 ± 14.6	0.718
Sex			
Male	3	6	0.256
Female	17	14	
ASA			
2	12	13	0.595
3	7	7	
4	1	0	
Comorbidity			
Diabetes Mellitus	6	5	0.723
Hypertension	5	8	0.311
Cerebral vascular accident	1	2	0.548
Chronic kidney disease	0	2	0.147
Chronic heart failure	1	0	0.311
Forearm and wrist vascular calcification	1	1	1.000
Fracture classification			
AO 2R3A	14	11	0.606
AO 2R3B	3	5	
AO 2R3C	3	4	
Operation time (minute)	57.8 ± 16.4	63.8 ± 20.6	0.512
Blood loss (mL)	14.2 ± 13.1	6.6 ± 5.9	**0.008**
Complication			
Infection	0	0	-
Neuropraxia	0	0	-
Compartment syndrome	0	0	-
Perioperative cardiovascular event	0	0	-
Hand vascular compromise event	1	0	0.331
Exchange to general anesthesia	0	-	-

WALANT, wide-awake local anesthesia no tourniquet; GA, general anesthesia; ASA, American Society of Anesthesiologists’ classification; mean ± standard deviation. Bold means *p*-value < 0.05.

The authors apologize for any inconvenience caused and state that the scientific conclusions are unaffected. This correction was approved by the Academic Editor. The original publication has also been updated.

## Figures and Tables

**Table 1:** Demographic data.

	WALANT	GA	*p*-Value
Patient number	20	20	
Age (year)	62.2 ± 13.5	59.9 ± 14.6	0.718
Sex			
Male	3	6	0.256
Female	17	14	
ASA			
2	12	13	0.595
3	7	7	
4	1	0	
Comorbidity			
Diabetes Mellitus	6	5	0.723
Hypertension	5	8	0.311
Cerebral vascular accident	1	2	0.548
Chronic kidney disease	0	2	0.147
Chronic heart failure	1	0	0.311
Forearm and wrist vascular calcification	1	1	1.000
Fracture classification			
AO 2R3B	14	11	0.606
AO 2R3C	3	5	
Blood loss (mL)	3	4	
Operation time (minute)	57.8 ± 16.4	63.8 ± 20.6	0.512
Blood loss (mL)	14.2 ± 13.1	6.6 ± 5.9	**0.008**
Complication			
Infection	0	0	-
Neuropraxia	0	0	-
Compartment syndrome	0	0	-
Perioperative cardiovascular event	0	0	-
Hand vascular compromise event	1	0	0.331
Exchange to general anesthesia	0	-	-

WALANT, wide-awake local anesthesia no tourniquet; GA, general anesthesia; ASA, American Society of Anesthesiologists’ classification; mean ± standard deviation. Bold means *p*-value < 0.05.
