# Supplementary material for: Less Fluctuation in Hemodynamics of the Wide-Awake Local Anesthesia No Tourniquet Technique Than General Anesthesia in Distal Radius Plating Surgery: A Prospective Case-Control Study
Source: J Clin Med. 2022 Feb 21;11(4):1123. doi: 10.3390/jcm11041123 (PMC8875852; doi:10.3390/jcm11041123)
Supplement: Supplementary file 1 [file jcm-11-01123-s001.zip › jcm-1579069-supplementary.pdf]

Supplementary Materials

Table S1 Perioperative mean arterial pressure and heart rate

	WALANT		GA		
	Mean	SD	Mean	SD	P-value*
**MAP**					
**T0**	105.36	13.40	104.17	18.70	0.819
**T1**	109.13	13.40	83.99	16.50	< 0.001
**T2**	103.84	16.57	78.81	22.39	< 0.001
**T3**	103.58	16.51	84.14	21.77	< 0.001
**T4**	103.41	18.92	81.17	14.05	< 0.001
**T5**	101.71	17.13	79.72	11.93	< 0.001
**T6**	102.17	17.40	78.40	11.90	< 0.001
**HR**					
**T0**	83.45	14.05	79.40	14.25	0.341
**T1**	84.35	15.00	74.85	12.56	0.028
**T2**	89.00	16.08	73.35	13.04	< 0.001
**T3**	89.85	15.20	68.95	11.30	< 0.001
**T4**	89.65	14.93	66.05	9.45	< 0.001
**T5**	87.65	15.03	68.10	10.30	< 0.001
**T6**	86.15	14.66	68.30	9.55	< 0.001

WALANT, wide-awake local anaesthesia no tourniquet; GA, general anaesthesia; MAP, mean arterial pressure (mmHg); HR, heart rate (beats per minute);

* the significance was adjusted for multiple comparisons with least significant difference (LSD)

Table S2 Pairwise comparisons in MAP and HR among WALANT group

Time		Mean Diff. in MAP	Std. Error	P-value*	Mean Diff. in HR	Std. Error	P-value*
T0	T1	-3.77	2.08	0.086	-0.90	1.88	0.638
	T2	1.52	2.38	0.531	-5.55	2.06	**0.014**
	T3	1.78	2.57	0.496	-6.40	2.22	**0.009**
	T4	1.95	2.99	0.522	-6.20	2.04	**0.007**
	T5	3.67	2.59	0.174	-4.20	2.22	0.073
	T6	3.20	2.71	0.252	-2.70	2.20	0.235
T1	T0	3.77	2.08	0.086	0.90	1.88	0.638
	T2	5.28	1.53	**0.003**	-4.65	1.97	**0.029**
	T3	5.55	1.90	**0.009**	-5.50	2.24	0.024
	T4	5.72	2.38	**0.027**	-5.30	2.13	**0.022**
	T5	7.43	1.95	**0.001**	-3.30	2.40	0.185
	T6	6.97	2.56	**0.014**	-1.80	2.69	0.511
T2	T0	-1.52	2.38	0.531	5.55	2.06	**0.014**
	T1	-5.28	1.53	**0.003**	4.65	1.97	**0.029**
	T3	0.27	0.98	0.789	-0.85	1.00	0.407
	T4	0.43	1.62	0.792	-0.65	0.92	0.486
	T5	2.15	1.33	0.123	1.35	1.84	0.471
	T6	1.68	1.98	0.406	2.85	2.06	0.182
T3	T0	-1.78	2.57	0.496	6.40	2.22	**0.009**
	T1	-5.55	1.90	**0.009**	5.50	2.24	**0.024**
	T2	-0.27	0.98	0.789	0.85	1.00	0.407
	T4	0.17	1.23	0.894	0.20	0.46	0.666
	T5	1.88	1.20	0.132	2.20	1.63	0.192
	T6	1.42	1.91	0.466	3.70	1.97	0.076
T4	T0	-1.95	2.99	0.522	6.20	2.04	**0.007**
	T1	-5.72	2.38	**0.027**	5.30	2.13	**0.022**
	T2	-0.43	1.62	0.792	0.65	0.92	0.486
	T3	-0.17	1.23	0.894	-0.20	0.46	0.666
	T5	1.72	1.44	0.246	2.00	1.49	0.196
	T6	1.25	2.04	0.548	3.50	1.80	0.066
T5	T0	-3.67	2.59	0.174	4.20	2.22	0.073
	T1	-7.43	1.95	**0.001**	3.30	2.40	0.185
	T2	-2.15	1.33	0.123	-1.35	1.84	0.471
	T3	-1.88	1.20	0.132	-2.20	1.63	0.192
	T4	-1.72	1.44	0.246	-2.00	1.49	0.196
	T6	-0.47	1.49	0.757	1.50	0.88	0.106
T6	T0	-3.20	2.71	0.252	2.70	2.20	0.235
	T1	-6.97	2.56	**0.014**	1.80	2.69	0.511
	T2	-1.68	1.98	0.406	-2.85	2.06	0.182
	T3	-1.42	1.91	0.466	-3.70	1.97	0.076
	T4	-1.25	2.04	0.548	-3.50	1.80	0.066
	T5	0.47	1.49	0.757	-1.50	0.88	0.106

WALANT, wide-awake local anaesthesia no tourniquet;

Diff., difference; Std., standard; * the significance was adjusted for multiple comparisons with least significant difference (LSD)

Table S3 Pairwise comparisons in MAP and HR among GA group

Time		Mean Diff. in MAP	Std. Error	P-value*	Mean Diff. in HR	Std. Error	P-value*
T0	T1	20.18	3.24	**< 0.001**	4.55	2.33	0.066
	T2	25.37	4.24	**< 0.001**	6.05	2.49	**0.025**
	T3	20.03	4.95	**0.001**	10.45	2.55	**0.001**
	T4	23.00	3.38	**< 0.001**	13.35	2.35	**< 0.001**
	T5	24.45	3.89	**< 0.001**	11.30	2.35	**< 0.001**
	T6	25.77	4.16	**< 0.001**	11.10	2.22	**< 0.001**
T1	T0	-20.18	3.24	**< 0.001**	-4.55	2.33	0.066
	T2	5.18	4.37	0.250	1.50	2.55	0.563
	T3	-0.15	4.22	0.972	5.90	2.72	**0.043**
	T4	2.82	2.94	0.350	8.80	2.29	**0.001**
	T5	4.27	3.29	0.211	6.75	2.56	**0.016**
	T6	5.58	3.49	0.126	6.55	2.37	**0.012**
T2	T0	-25.37	4.24	**< 0.001**	-6.05	2.49	**0.025**
	T1	-5.18	4.37	0.250	-1.50	2.55	0.563
	T3	-5.33	3.12	0.104	4.40	1.39	**0.005**
	T4	-2.37	3.08	0.451	7.30	1.70	**< 0.001**
	T5	-0.92	4.15	0.828	5.25	1.88	**0.011**
	T6	0.40	4.50	0.930	5.05	1.92	**0.017**
T3	T0	-20.03	4.95	**0.001**	-10.45	2.55	**0.001**
	T1	0.15	4.22	0.972	-5.90	2.72	**0.043**
	T2	5.33	3.12	0.104	-4.40	1.39	**0.005**
	T4	2.97	2.67	0.280	2.90	0.96	**0.007**
	T5	4.42	3.70	0.247	0.85	1.60	0.601
	T6	5.73	3.76	0.144	0.65	1.49	0.668
T4	T0	-23.00	3.38	**< 0.001**	-13.35	2.35	**< 0.001**
	T1	-2.82	2.94	0.350	-8.80	2.29	**0.001**
	T2	2.37	3.08	0.451	-7.30	1.70	**< 0.001**
	T3	-2.97	2.67	0.280	-2.90	0.96	**0.007**
	T5	1.45	1.89	0.452	-2.05	1.42	0.166
	T6	2.77	1.99	0.180	-2.25	0.97	**0.031**
T5	T0	-24.45	3.89	**< 0.001**	-11.30	2.35	**< 0.001**
	T1	-4.27	3.29	0.211	-6.75	2.56	**0.016**
	T2	0.92	4.15	0.828	-5.25	1.88	**0.011**
	T3	-4.42	3.70	0.247	-0.85	1.60	0.601
	T4	-1.45	1.89	0.452	2.05	1.42	0.166
	T6	1.32	1.21	0.288	-0.20	0.99	0.843
T6	T0	-25.77	4.16	**< 0.001**	-11.10	2.22	**< 0.001**
	T1	-5.58	3.49	0.126	-6.55	2.37	**0.012**
	T2	-0.40	4.50	0.930	-5.05	1.92	**0.017**
	T3	-5.73	3.76	0.144	-0.65	1.49	0.668
	T4	-2.77	1.99	0.180	2.25	0.97	**0.031**
	T5	-1.32	1.21	0.288	0.20	0.99	0.843

GA, general anaesthesia; Diff., difference; Std., standard; * the significance was adjusted for multiple comparisons with least significant difference (LSD)
